# Histone Methylation Marks on Circulating Nucleosomes as Novel Blood-Based Biomarker in Colorectal Cancer

**DOI:** 10.3390/ijms161226180

**Published:** 2015-12-11

**Authors:** Ugur Gezer, Ebru E. Yörüker, Metin Keskin, Cemil Burak Kulle, Yoganiranjana Dharuman, Stefan Holdenrieder

**Affiliations:** 1 Department of Basic Oncology, Oncology Institute, Istanbul University, Istanbul 34093, Turkey; ebruyoruker@gmail.com; 2 Department of General Surgery, Istanbul Medical Faculty, Istanbul University, Istanbul 34093, Turkey; mtnkeskin@yahoo.com (M.K.); cemilburakkulle@gmail.com (C.B.K.); 3 Institute of Clinical Chemistry and Clinical Pharmacology, University of Bonn, Bonn D-53127, Germany; dharuman@hotmail.com (Y.D.); stefan.holdenrieder@uni-bonn.de (S.H.)

**Keywords:** nucleosomes, histone modification, immunoassay, colorectal cancer

## Abstract

Circulating nucleic acids (CNAs) are under investigation as a liquid biopsy in cancer as potential non-invasive biomarkers, as stable structure in circulation nucleosomes could be valuable sources for detection of cancer-specific alterations in histone modifications. Our interest is in histone methylation marks with a focus on colorectal cancer, one of the leading cancers respective the incidence and mortality. Our previous work included the analysis of trimethylations of lysine 9 on histone 3 (H3K9me3) and of lysine 20 on histone 4 (H4K20me3) by chromatin immuno- precipitation-related PCR in circulating nucleosomes. Here we asked whether global immunologic measurement of histone marks in circulation could be a suitable approach to show their potential as biomarkers. In addition to H3K9me3 and H4K20me3 we also measured H3K27me3 in plasma samples from CRC patients (*n* = 63) and cancer free individuals (*n* = 40) by ELISA-based methylation assays. Our results show that of three marks, the amounts of H3K27me3 (*p* = 0.04) and H4K20me3 (*p* < 0.001) were significantly lower in CRC patients than in healthy controls. For H3K9me3 similar amounts were measured in both groups. Areas under the curve (AUC) in receiver operating characteristic (ROC) curves indicating the power of CRC detection were 0.620 for H3K27me3, 0.715 for H4K20me3 and 0.769 for the combination of both markers. In conclusion, findings of this preliminary study reveal the potential of blood-based detection of CRC by quantification of histone methylation marks and the additive effect of the marker combination.

## 1. Introduction

Despite intensive research and significant advancements in treatment options, colorectal cancer (CRC) is still one of the leading cancers respective incidence and mortality [1]. Even if advances in screening programs, surgical techniques, medical treatment and surveillance programs led to improved survival rates of CRC patients [2], CRC patient survival is still poor, especially in advanced disease. Despite many potential biomarkers have been described, the number of biomarkers that have been integrated into clinical practice is small [3]. On the other hand, several genetic and epigenetic pathways have been shown to be involved in determining patient prognosis and survival of CRC patients [4].

Through research in last two decades it has become increasingly evident that altered epigenetic control of gene expression plays an important role in carcinogenesis [5]. Epigenetic biomarkers are increasingly recognized as promising diagnostic and prognostic tools in CRC [6]. Along with non-coding RNAs, DNA methylation and chromatin remodeling, histone modifications (HMs) are an essential part of epigenetic pathway. HMs post-translationally occur at highly dynamic N-terminal amino acid tails of 20–35 residues in length extending from the surface of nucleosome, the basic unit of eucaryotic chromatin packaging. Given histone residues on these tails can post-translationally become methylated, phosphorylated, acetylated, *etc.* In an interplay with DNA methylation, HMs regulate the accessibility of chromatin to gene expression. Combinatorial effect of different HMs defined as “histone code” [7] plays a pivotal role in eukaryotic gene regulation and in fine folding of nucleosomes into higher order chromatin [8].

Since in cancer patients elevated release of nucleosomes and DNA into blood circulation occurs due to increased cell turnover [9], nucleosomes as stabile structures in circulation [10] could provide valuable sources of novel epigenetic biomarkers. Studies based on the measurement of amounts of circulating nucleosomes in plasma and serum revealed that circulating nucleosomes are generally found in increased amounts in blood of cancer patient. However, various benign diseases are also often associated with an elevated serum level of nucleosomes limiting its diagnostic value [11].

Detection of cancer-specific HMs in circulating nucleosomes could represent a novel type of biomarkers. Our previous work based chromatin immunoprecipitation (ChIP)-based quantitative PCR or sequencing suggests that trimethylations of lysine 9 on histone 3 (H3K9me3) and of lysine 20 on histone 4 (H4K20me3), the hallmarks of pericentric heterochromatin [12], are found at decreased amounts in circulating nucleosomes in CRC patients compared to healthy individuals or some other malignancies [13,14,15]. Characterization of HMs in circulating nucleosomes by ChIP assay is a tedious process. In the present study, we aimed to measure histone lysine methylation in circulating nucleosomes globally and adapted therefore commercially available ELISA assays for their use in serum and plasma and measured histone lysine methylation in new cohort of CRC patients. In addition to H3K9me3 and H4K20me3, we also measured another repressive methylation mark, H3K27me3.

## 2. Results

**Figure 1:**
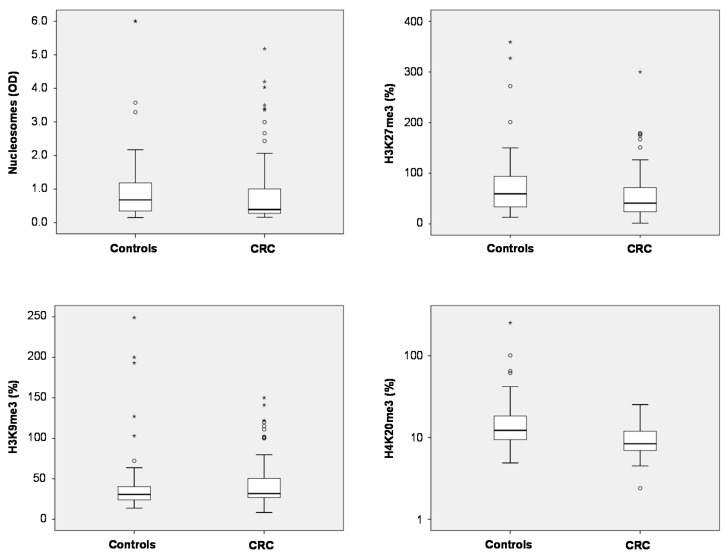
Box plots indicating median, interquartile ranges, whiskers and ranges for nucleosomes, H3K9me3, H3K27me3 and H4K20me3 in patients with colorectal cancer (CRC) and non-malignant controls.

Total nucleosome levels measured by ELISA showed a trend to lower levels in plasma of CRC patients as compared with non-malignant controls (Table S1) (medians 0.39 *vs.* 0.67; *p* = 0.113). For the three methylation marks, significant differences were found for H3K27me3 and H4K20me3: In CRC patients, median plasma levels of H3K27me3 were lower than in controls (41.0% *vs.* 59.4%; *p* = 0.041). Similarly, H4K20me3 median levels were found to be lower in plasma CRC patients than in controls (8.4% *vs.* 12.3%; *p* < 0.001). For H3K9me3, there was no difference between both groups (medians CRC 31.7%, controls 30.8%; *p* > 0.05) (Figure 1).

The various histone marks significantly (*p* < 0.001) correlated with each other with correlation coefficients between 0.62 and 0.65. Combination of the histone marks H4K20me3 and H3K27me3 as well as H4K20me3 and H3K9me3 achieved significant differences between the CRC and control groups, too (both *p* < 0.001).

As Table 1 illustrates, measured parameters correlate with some clinicopathological variables of CRC patients. Circulating nucleosomes are higher in patients ≥60 years (*p* = 0.01). Regarding histone methylation marks, H4K20me3 levels were higher in plasma of patients with lymphocyte invasion (*p* = 0.03) and in those with Crohn’s-like disease (*p* = 0.01).

The power of discrimination between two groups is best shown by receiver operating characteristic (ROC) curves that mirror the profile of sensitivity and specificity over the whole range of possible cutoffs. The area under the curve (AUC) and sensitivities at a defined specificity (e.g., 95% or 90%) of the control groups are measures to compare various markers with each other.

**Table 1:** Association of nucleosomes, H3K27me3, H3K9me3, and H4K20me3 with clinical characteristics.

Variables	Number of Patients (%)	Nucleosomes (OD)		H3K27me3 (%)		H3K9me3 (%)		H4K20me3 (%)	
		Median	*p*-Value	Median	*p*-Value	Median	*p*-Value	Median	*p*-Value
**Age**									
≥60 years	30 (47.6)	0.43	0.01	54.55	0.25	29.70	0.69	8.20	0.72
<60 years	33 (52.3)	0.31		36.10		33.10		8.40	
**Gender**									
Male	36 (57.1)	0.39	0.99	51.55	0.54	29.70	0.45	10.25	0.29
Female	27 (42.8)	0.39		36.10		34.70		8.20	
**Diameter**									
≥5 cm	32 (50.7)	0.42	0.75	51.55	0.82	37.40	0.22	9.55	0.10
<5 cm	31 (49.2)	0.36		36.10		29.30		7.80	
**Differentiation**									
Well + Moderate	49 (77.7)	0,37	0.42	38.90	0.52	31.30	0.90	8.20	0.25
Poor	14 (22.2)	0.42		58.10		36.20		9	
**Lymphatic Metastasis**									
N0	30 (47.6)	0.40	0.89	49.65	0.90	30.90	0.73	8.30	0.75
N1-3	33 (52.3)	0.37		37.20		32.10		8.40	
**Distal Metastasis ^a^**									
M0	52 (85.2)	0.40	0.59	40	0.54	30.25	0.54	8.35	0.75
M1	9 (14.7)	0.44		33.4		38.40		8.30	
**TNM Stage**									
I–II	28 (44.4)	0.42	0.41	49.65	0.59	30.90	0.92	8.30	0.76
III–IV	35 (55.5)	0.36		37.20		32.10		8.40	
**Tumor Localization**									
Right	27 (42.8)	0.41	0.94	48.70	0.74	33.10	0.37	10.20	0.29
Left	36 (57.1)	0.37		39.95		30.70		8.25	
**Venous Invasion ^b^**									
No	46 (80.7)	0.41	0.80	38.95	0.87	30.05	0.30	8.50	0.15
Yes	11 (19.2)	0.37		66.50		37.40		8.20	
**Crohn’s-like ^c^**									
No	39 (69.6)	0.37	0.67	33.40	0.25	29.30	0.19	8.30	0.01
Yes	17 (30.3)	0.42		48.70		34.70		11.70	
**Lymphocyte Invasion ^c^**									
No	44 (78.5)	0.4	0.06	46.80	0.28	29.7	0.33	8.45	0.03
Yes	12 (21.4)	0.3		34.75		36.1		14.45	
**Perineural Invasion**									
No	38 (67.8)	0.40	0.40	41.95	0.81	30.05	0.45	8.40	0.93
Yes	18 (32.1)	0.36		32.20		36.70		9	
**Mesenteric Tumor Nodules ^c^**									
No	38 (67.8)	0.40	0.81	35.95	0.22	31.60	0.69	8.50	0.90
Yes	18 (32.1)	0.33		55.75		30.85		8.90	
**CEA ^a^**									
≤5 ng/mL	35 (57.3)	0.36	0.63	41	0.76	32.1	0.87	8.30	0.74
>5 ng/mL	26 (42.6)	0.41		51.55		32.7		8.55	
**CA 19-9 ^a^**									
≤34 U/mL	51 (83.6)	0.39	0.86	41	0.70	31.70	0.26	8.20	0.17
>34 U/mL	10 (16.3)	0.39		51.7		37.35		10.60	

^a^ Not available for 2 patients; ^b^ Not available for 6 patients; ^c^ Not available for 7 patients. Bold p values indicate statistical significance.

**Figure 2:**
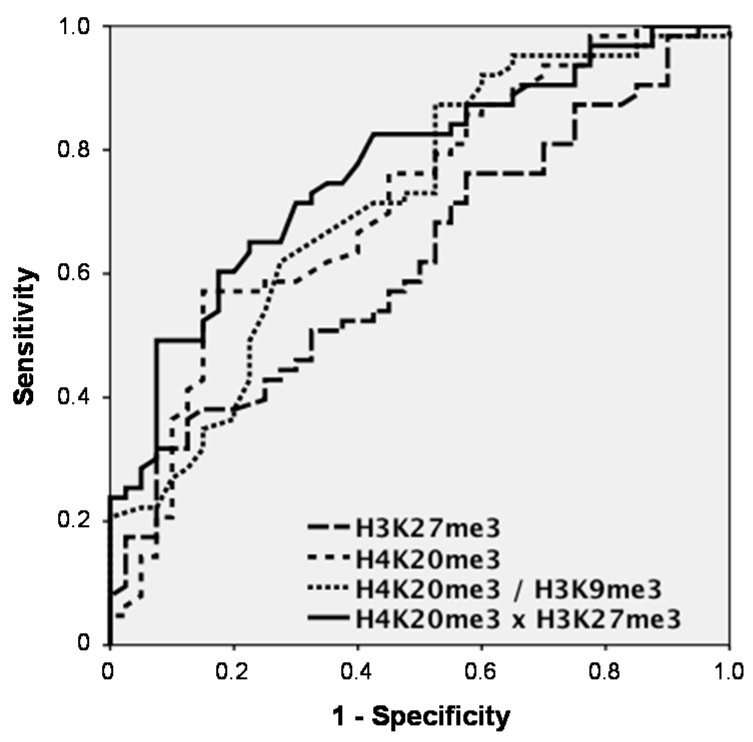
Receiver operating characteristic (ROC) curves indicating the sensitivity-specificity profile for the discrimination between patients with colorectal cancer (CRC) and non-malignant controls over the whole range of possible cutoffs for the significantly discriminating marks H3K27me3 and H4K20me3, as well as for the combinations H4K20me3/H3K9me3 and H4K20me3 × H3K27me3 determined by ELISA.

Among the significant discriminating markers, H3K27me3 achieved an AUC of 0.620 (95% confidence interval (CI) = 0.512–0.728) and sensitivities for CRC detection of 17.5% at 95% and 31.7% at 90% specificity *vs.* controls, while H4K20me3 had an AUC of 0.715 (CI = 0.613–0.818) and a sensitivity of 14.3% at 95% and of 36.5% at 90% specificity. When combining histone marks, H4K20me3 and H3K9me3 did not increase the AUC (0.714; CI = 0.612–0.816). However, H4K20me3 and H3K27me3 clearly showed superior results with an AUC of 0.769 (CI = 0.679–0.860) and sensitivities of 28.6% at 95% and of 49.2% at 90% specificity (Figure 2).

Upon ELISA findings, we performed a ChIP-PCR assay including H3K9me3 and H4K20me3 in a subset of samples. We randomly selected 50 samples from the patients and 36 from cancer-free individuals. In this subset, both methods did not correlate with each other, neither for H3K9me3 nor for H4K20me3 (*p* > 0.05). Further, we did not find significant differences between the patient groups for either mark nor for the ratio of both marks when using the ChIP-PCR method. However, there was a trend to lower H4K20me3/H3K9me3 ratios in CRC patients (*p* = 0.090).

## 3. Discussion

Circulating nucleic acids (CNAs) are under investigation as a liquid biopsy in cancer as several aspects of CNAs including aberrant DNA methylation, microRNAs or amounts of DNA and mRNA have been shown to bear the potential as useful cancer biomarker [16]. Circulating nucleosomes are stabile structures and could serve as valuable source of novel serum and plasma biomarkers [17]. Our previous work including H3K9me3 and H4K20me3 suggest histone methyl marks as candidate biomarkers in CRC [13,14,15]. Here we aimed to demonstrate whether global measurement of histone marks in circulation could be a suitable approach to characterize them as potential biomarkers. While serum may show higher amounts of CNA than plasma [18], we here used plasma for this clinical study.

In methodical analysis of the pretesting phase we showed that histone marks could generally be measured in a reproducible way by ELISA technique. However, as these assays were developed for research use only (RUO), optimization work is necessary to bring them into a high-quality version that can be applied for patient diagnostic later. Our clinical investigations revealed that of three methylation marks, the amounts of H3K27me3 and H4K20me3 were lower in plasma of CRC patients than in cancer-free individuals. Unlike earlier ChIP-based findings, H3K9me3 was found in comparable levels in plasma of both study groups. In contrast, H4K20me3 findings were in line with earlier ChIP-based studies in which we amplified Sat II on modified nucleosomes and found lower levels in CRC patients. It has to be emphasized that in these previous studies, the levels of H3K9me3 were stronger reduced in plasma samples from Turkish colorectal cancer patients [13] while in an other study on German samples both marks were significantly lower in CRC samples [14]. In the subsequent study including the sequencing of immunoprecipitated fragments we found decreases for both marks. These differences by use of the two ChIP- and ELISA-based methods are not surprising: in the first one, a specific region of the genome is amplified, while ELISA assay measures methylation levels globally.

Even if ChIP assay and ELISA assay did not correlate with each other in our study, H3K9me3 and/or H4K20me3 appear to be of clinical significance in CRC and other cancers. An early study described the loss of H4K20me3 as a common hallmark of human tumor cells [19]. A recent retrospective study conducted by immunohistochemistry in archived tumor samples demonstrated that low expression of H3K9me3 and H4K20me3 are correlated with shorter patient survival and higher chances of tumor recurrence in early-stage colon cancer [20]. These findings are in line with our results as we found low H4K20me3 levels being associated with tumors without lymphocyte invasion and inflammatory features (Crohn’s like disease), which are well-known indicators of poor prognosis [21]. Similarly, loss of H4K20 trimethylation in breast or lung tumors has been found to affect prognosis of patients of these cancers [22,23]. This data suggests that loss of H4K20me3 might be a global event in cancer that we also could detect in blood circulation of CRC patients.

Concerning power of discrimination, H4K20me3 showed to be the most meaningful marker in this setting, followed by H3K27me3. Impressively, the combination of these two marks showed additive sensitivity and increased AUCs in ROC curves. While these results on histone marks in blood are quite promising, further studies have to be added with higher numbers of patients, additional control groups like patients with benign colorectal diseases and improved test settings.

## 4. Materials and Methods

### 4.1. Patient Cohorts

We have enrolled 63 patients with colorectal cancer and 40 control individuals into the study. Samples were collected between July 2013 and August 2014 from the Surgical Department of Istanbul Medical University Hospital. Blood specimens were drawn from the patients prior to surgery or any medical treatment. The control group included individuals who underwent colonoscopy and were verified to be cancer- and polyp-free and age-matched to patients. The control group comprised 16 individuals undergoing screening exams, four individuals with family history (but no signs) of colorectal cancer, seven with abdominal pain, 10 with bleeding and three with constipation (Table S1). Patients’ clinicopathological characteristics are given in Table 1. Levels of histone marks were correlated with the clinical variables including age and gender, tumor diameter, TNM stage, differentiation, lymphatic and distant metastasis status, venous invasion or the tumor markers CEA and CA19-9 (Abcam, Cambridge, MA, USA). The study was approved by the Istanbul Faculty of Medicine Ethics Committee and informed consent was obtained from participants.

Preanalytics: After venous puncture, centrifugation was done within 2 h at 1800 rpm (Hettich, Tuttlingen, Germany) at a for 30 min. Subsequently, plasma samples were stored at −80 °C until measurement.

### 4.2. Quantitation of Circulating Nucleosomes

The concentrations of circulating nucleosomes in serum and plasma were determined using the Cell-Death Detection ELISA plus (Roche Diagnostics, Mannheim, Germany) as reported previously [24]. Briefly, we applied twenty microliters of samples in duplicate, and mean absorbances at 405 nm (reference wavelength approximately 490 nm) were used to calculate the relative serum concentrations.

### 4.3. Histone Methylation ELISA Assays

EpiQuik Global Tri-Methyl Histone Quantification Kits (Epigentek, Farmingdale, NY, USA) were employed to analyze and measure H3K9me3, H3K27me3 and H4K20me3 in blood circulation. The protocol of the kit was adapted for use with blood plasma in which we directly applied plasma samples instead of the extraction of histones. Extracellular nucleosomes bearing those modifications are captured to the strip wells coated with corresponding anti-tri-methyl antibodies. The captured tri-methylated histones were then detected with a labeled detection antibody, followed by a color development reagent. The ratio of methylation is proportional to the intensity of absorbance.

We applied 25 µL plasma as duplicates and the measurements were done according to the instructions of the manufacturer. Absorbance was measured at 450 nm. As exact amounts of nucleosomes are not known in plasma, instead of calculating absolute protein concentrations we utilized methylation percentage for each sample through following equation: tri-methylation % = (OD test sample − blank) × 100.

To minimize interassay variability, cases and controls were mixed in all plates and some patient plasma samples were used as controls over all plates.

In a methodical pretesting phase, we determined the intra- and inter-assay variation of histone methylation assays. We found an intraassay coefficient of variation (CV) of 7.0%, 9.4% and 19.8% for H3K27me3, H3K9me3 and H4K20me3, respectively. Regarding inter-assay comparison, CVs were 7.8%, 8.8%, and 19.5% for H3K27me3, H3K9me3 and H4K20me3, respectively. For both evaluations, levels of H4K20me3 were considerably lower (mean level 15%) as compared with H3K27me3 (mean level 55%) and H3K9me3 (mean level 42%) explaining the high CVs found for H4K20me3.

### 4.4. Chromatin Immunoprecipitation Assay

For ChIP assay we used the ChromaFlash™ High-Sensitivity ChIP Kit (Epigentek, Farmingdale, NY, USA). The protocol of the kit was adapted for the use with blood plasma to allow the capture of low abundance methylated histones. No chromatin extraction and sharing was made. We applied 25 μL of plasma for the assay and followed kit instructions. Briefly, we set up the ChIP reactions by adding ChIP buffer, plasma, enrichment enhancer (2 μL), BS blocker solution (10 μL) to a total of 100 μL to the well of strips and incubated over night at +4 °C on an orbital shaker (100 rpm). In addition to specific antibodies, we also included a positive control antibody (RNA polymerase II), and a negative control (non-immune IgG). After several wash steps, precipitated chromatin fragments were treated with RNase A (Roche Diagnostics) (at 42 °C for 30 min) and Proteinase K (Roche Diagnostics) (at 60 °C for 45 min). DNA was then transferred into PCR tubes and incubated in a thermocycler at 95 °C (Thermo Fisher Scientific, Wlatham, MA, USA) for 15 min. Subsequently, DNA was transferred to purification columns. After washing steps, DNA was eluted in 30 μL DNA elution buffer and stored at −20 °C until use.

### 4.5. Quantitative PCR

Based on our previous findings [13,14,15], in quantitative PCR (qPCR) we amplified satellite II (Sat II) elements. Primer sequences (Integrated DNA technologies, Coralville, IA, USA) used for Sat II were 5′-CATCGAATGGAAATGAAAGGAGTC-3’ (F) and 5′-ACCATTGGATGATTGCAGTCAA-3’ (R) [25].

Three-microliter (out of 30 µL, precipitated from 200 µL plasma) ChIP-DNA were subjected to qPCR, which was performed in the LightCycler 480 Instrument (Roche Diagnostics) using SYBR Green I (Roche Diagnostics) as the fluorescence molecule. We used a gradual PCR program with annealing temperature starting at 60 °C for 2 cycles followed by 38 cycles at 55 °C. Samples with a threshold cycle (*C*_t_) >40 were considered negative. Amplification of the appropriate product was confirmed by melting curve analysis following amplification. To estimate the amounts of H3K9me3- and H4k20me3-related Sat II sequences in immune precipitated fragments, we subtracted non-Ig values from qPCR values of each sample and performed an absolute quantification using a dilution series of a sample with known DNA concentration.

### 4.6. Statistics

Results were presented as median, quartiles and total ranges. Correlations between parameters were calculated by the Pearson test. Discrimination between the patient group and cancer-free control subjects was done by the Mann–Whitney test. Sensitivities are given at 95% specificity for tumor detection. Further receiver operating characteristic (ROC) curves and areas under the curves (AUC) were calculated to test the sensitivity-specificity profile over all possible cutoffs. Correlations between both methods were tested by Spearman’s rank correlation test. A *p*-value <0.05 was considered statistically significant. Calculations were performed using the statistical software SPSS 21.0 (IBM Corporation, Armonk, NY, USA).

## 5. Conclusions

Our findings provided by ELISA-based histone methylation assays reveal that H4K20me3 and H3K27me3 on circulating nucleosomes are reduced in plasma of CRC patients compared to cancer-free individuals and confirm, in part, our previous data, obtained by ChIP assay. As ELISA technique has advantages in cost, time, and labor over others, it may be an appropriate approach to detect global changes in histone modifications and to develop assays for serial measurements. Our findings also suggest that the combination of several histone marks will conclusively enhance sensitivity and specificity of cancer detection. The findings of this pilot study need to be confirmed in further prospective trials with larger cohorts. However, it represents a first hint for histone methylation marks as promising biomarkers in CRC.

## Supplementary Materials

Supplementary materials can be found at http://www.mdpi.com/1422-0067/16/12/26180/s1.
