# Supplementary material for: Histone Methylation Marks on Circulating Nucleosomes as Novel Blood-Based Biomarker in Colorectal Cancer
Source: Int J Mol Sci. 2015 Dec 11;16(12):29654–62. doi: 10.3390/ijms161226180 (PMC4691123; doi:10.3390/ijms161226180)
Supplement: Supplementary file 1 [file ijms-16-26180-s001.pdf]

Supplementary Materials: Histone Methylation Marks on Circulating Nucleosomes as Novel Blood-Based Biomarker in Colorectal Cancer

Ugur Gezer, Ebru E. Yörüker, Metin Keskin, Cemil Burak Kulle, Yoganiranjana Dharuman and Stefan Holdenrieder

Table S1. Characteristics of the controls.

Control Group	History	Gender	Age
C1	Bleeding	F	60
C2	Abdominal pain	M	61
C3	Bleeding	F	41
C4	Family History (+)	M	59
C5	Abdominal pain	M	54
C6	Abdominal pain	F	52
C7	Screening	F	60
C8	Screening	F	40
C9	Bleeding	M	35
C10	Bleeding	M	58
C11	Constipation	F	38
C12	Constipation	M	38
C13	Bleeding	F	63
C14	Abdominal pain	F	59
C15	Bleeding	M	38
C16	Bleeding	F	84
C17	Screening	M	44
C18	Family History (+)	M	44
C19	Family History (+)	M	44
C20	Screening	F	56
C21	Screening	F	59
C22	Screening	M	45
C23	Screening	F	49
C24	Screening	F	58
C25	Screening	M	63
C26	Bleeding	M	57
C27	Screening	M	39
C28	Family History (+)	M	55
C29	Screening	M	63
C30	Abdominal pain	M	54
C31	Abdominal pain	F	46
C32	Screening	F	47
C33	Screening	F	44
C34	Screening	M	38
C35	Screening	M	59
C36	Abdominal pain	M	64
C37	Screening	M	49
C38	Bleeding	W	54
C39	Bleeding	M	39
C40	Constipation	M	52
